# Supplementary material for: Causal Relationship between Aging and Anorexia Nervosa: A White-Matter-Microstructure-Mediated Mendelian Randomization Analysis
Source: Biomedicines. 2024 Aug 16;12(8):1874. doi: 10.3390/biomedicines12081874 (PMC11351342; doi:10.3390/biomedicines12081874)
Supplement: Supplementary file 1 [file biomedicines-12-01874-s001.zip › Supplementary Figure/Figure S8/Leaveoneout plot of MR analysis for TL to ubm-b-1530.pdf]

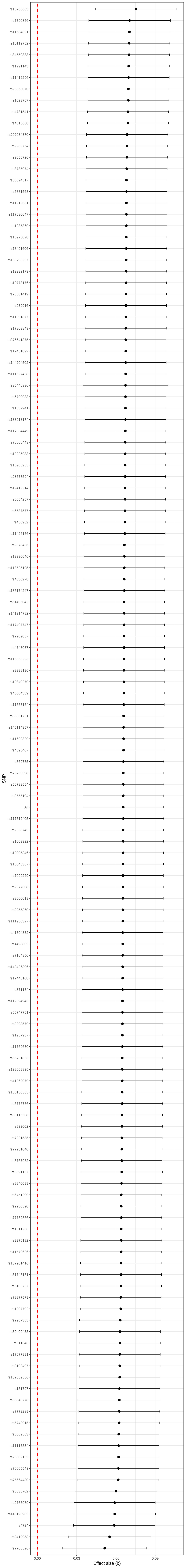

SNP
Effect size (b)
0.00
0.03
0.06
0.09
rs10768683
rs7790856
rs11584821
rs10112752
rs34550383
rs1291143
rs11412296
rs28363070
rs1023767
rs4731541
rs4616688
rs202034370
rs2282764
rs2056726
rs3785074
rs80324517
rs6881568
rs11212631
rs117630647
rs1985369
rs16978028
rs78491606
rs139795227
rs12932179
rs10773176
rs73581419
rs939916
rs11991877
rs17803849
rs376641875
rs12451892
rs144204502
rs111527438
rs35446936
rs6790988
rs1332941
rs188918174
rs117034449
rs76666449
rs12925933
rs10905255
rs28577594
rs12412214
rs6054257
rs6587577
rs450962
rs11426156
rs9878436
rs13230646
rs113525195
rs4530278
rs185174247
rs61405042
rs141214782
rs117407747
rs7209057
rs4743037
rs116863223
rs9398196
rs10840270
rs45604339
rs11557154
rs56061761
rs145114957
rs11699829
rs4695407
rs869785
rs73730598
rs56799554
rs2555104
A8
rs117512405
rs2538745
rs1003322
rs10805346
rs10845387
rs7099229
rs2977608
rs9600019
rs9955360
rs111950327
rs41304632
rs4498805
rs7164950
rs142426306
rs17445108
rs871134
rs112394943
rs55747751
rs2293579
rs1957937
rs11769630
rs66731853
rs139669835
rs41269078
rs150150565
rs6776756
rs80116508
rs932002
rs7221585
rs77231040
rs3767952
rs3891167
rs9940099
rs6751209
rs2230590
rs77732866
rs1611236
rs2276182
rs11579626
rs137901416
rs61748181
rs8105767
rs79977579
rs1907702
rs2967355
rs59409453
rs611646
rs17677991
rs8102497
rs182059586
rs131797
rs35640778
rs7772289
rs5742915
rs6669563
rs11117354
rs28502153
rs76065543
rs75664430
rs6536702
rs2763979
rs143190905
rs4724
rs9419958
rs7705526
